# Efficacy of inspiratory muscle training on weaning success in mechanically ventilated ICU patients: a systematic review and network meta-analysis of randomized controlled trials

**DOI:** 10.1186/s12890-026-04220-3

**Published:** 2026-03-27

**Authors:** Erika Meléndez-Oliva, Juan Nicolás Cuenca-Zaldívar, Nina Cadeau Comte, Eleuterio A. Sánchez-Romero, Rob Sillevis, Camilo Corbellini

**Affiliations:** 1 Interdisciplinary Research Group on Musculoskeletal Disorders, Madrid, 28670 Spain; 2 Department of Optics, Pharmacology and Anatomy. Grupo de Investigación en Dietética Aplicada, Nutrición y Composición Corporal (DANuC), University of Alicante, Alicante, 03690 Spain; 3 Physiotherapy and Orofacial Pain Working Group, Sociedad Española de Disfunción Craneomandibular y Dolor Orofacial (SEDCYDO), Madrid, 28009 Spain; 4 Research Group in Nursing and Health Care, Puerta de Hierro Health Research Institute-Segovia de Arana (IDIPHISA), Majadahonda, 28222 Spain; 5 Physical Therapy Unit, Primary Health Care Center El Abajón, Las Rozas de Madrid, 28231 Spain; 6 Jean Jaurès, Toulouse, France; 7 Department of Rehabilitation Sciences, Florida Gulf Coast University, Fort Myers, FL 33965 USA; 8 Physiotherapy Masters of Science Programme, LUNEX International University of Health, Exercise and Sports, 50, Avenue du Parc des Sports, Differdange, 4671 Luxembourg; 9 Department of Physiotherapy, LUNEX International University of Health, Exercise and Sports, Differdange, Luxembourg; 10 Luxembourg Health and Sport Sciences Research Institute A.s.b.l, Differdange, Luxembourg

**Keywords:** Inspiratory muscle training, Mechanical ventilation, ICU, Weaning, Maximal inspiratory pressure, RSBI, Systematic review, Network meta-analysis

## Abstract

**Background:**

Inspiratory muscle training (IMT) has been proposed as a strategy to mitigate ventilator-induced diaphragmatic dysfunction in mechanically ventilated intensive care unit (ICU) patients. However, its comparative effectiveness across different training intensities and impact on weaning success remain uncertain.

**Objective:**

To evaluate the efficacy of intima-media thickness (IMT) on weaning success, maximal inspiratory pressure (MIP), and rapid shallow breathing index (RSBI) in mechanically ventilated adult intensive care unit (ICU) patients.

**Methods:**

This systematic review followed the Preferred Reporting Items for Systematic Reviews and Meta-Analyses (PRISMA) 2020 guidelines and was prospectively registered in the International Prospective Register of Systematic Reviews (PROSPERO; CRD420251026681). PubMed, Cochrane Library, and PEDro were searched through July 2025 for randomized controlled trials (RCTs) including ICU adults receiving invasive mechanical ventilation for ≥ 48 h. The primary outcome measure was successful weaning. The secondary outcomes were MIP and RSBI. The Risk of bias was assessed using RoB 2.0. A frequentist network meta-analysis was conducted using standardized mean differences (SMD) and odds ratios (OR).

**Results:**

Eighteen RCTs (*n* = 1137) were included. Network meta-analysis demonstrated that high-intensity IMT (HI-IMT) and HI-IMT combined with proprioceptive neuromuscular facilitation showed significant improvements in MIP compared to controls. No significant differences were observed between the active interventions for RSBI. For weaning success, the HI-IMT group showed a higher probability of successful weaning than the control group (odds ratio [OR] 0.32, 95% CI 0.14–0.75). Heterogeneity was substantial for continuous outcomes (I² >85%) and negligible for weaning success (I²=0%).

**Conclusions:**

IMT improves inspiratory muscle strength in mechanically ventilated ICU patients, particularly when delivered at higher intensities. Although high-intensity IMT was associated with improved weaning success in the network analysis, the overall clinical impact should be interpreted cautiously, given the heterogeneity and indirect nature of some comparisons. Further adequately powered trials with standardized protocols are warranted.

**Supplementary Information:**

The online version contains supplementary material available at 10.1186/s12890-026-04220-3.

## Introduction

Mechanical ventilation (MV) is a life-saving intervention for patients with acute or acute-on-chronic respiratory failure admitted to the intensive care unit (ICU) [1, 2]. However, MV exposure is consistently associated with clinically relevant adverse effects including ventilator-induced diaphragm dysfunction, respiratory muscle disuse atrophy, and global inspiratory muscle weakness [3–5]. Respiratory muscle weakness has been reported in up to 60–65% of critically ill patients and appears to be approximately twice as prevalent as peripheral muscle weakness, reflecting the combined effects of mechanical unloading, systemic inflammation, sedation, and neuromuscular impairment [3–5]. Respiratory dysfunction has been independently associated with prolonged MV duration, extubation failure, ICU-acquired weakness, and increased short- and long-term morbidity and mortality [3, 4, 6].

Effective liberation from mechanical ventilation depends on the ability of the diaphragm and accessory inspiratory muscles to generate sufficient inspiratory pressure while avoiding an excessive respiratory load [6, 7]. Diaphragmatic dysfunction is highly prevalent in mechanically ventilated patients and has been consistently associated with prolonged weaning, extubation failure, and increased mortality [3, 6, 7]. Inspiratory muscle training aims to counteract ventilator-induced diaphragmatic weakness by improving maximal inspiratory pressure and reducing the rapid shallow breathing pattern, thereby enhancing the physiological reserve required for successful weaning [8–10].

Weaning from MV, defined as the gradual reduction and eventual withdrawal of ventilatory support, is a complex and critical phase of ICU management that requires an adequate balance between respiratory muscle capacity and the mechanical load imposed on the respiratory system [1, 6]. In routine clinical practice, weaning is most commonly performed using spontaneous breathing trials (SBTs) delivered through techniques such as T-piece trials, continuous positive airway pressure (CPAP), pressure support ventilation (PSV), or automatic tube compensation [1, 2]. Readiness for weaning is typically assessed using a combination of physiological parameters, including respiratory rate (RR), tidal volume (VT), maximal inspiratory pressure (MIP), and the rapid shallow breathing index (RSBI or f/VT), which aim to capture respiratory muscle strength, endurance, and ventilatory efficiency [9–13]. Frequently applied threshold values include RR ≤ 35 breaths·min⁻¹, VT > 5 mL·kg⁻¹, RSBI < 105 breaths·min⁻¹·L⁻¹, and MIP ≤–20 to − 25 cmH₂O, although the diagnostic and prognostic performance of these cut-offs varies across patient populations and clinical settings [9, 11, 12].

Successful completion of an SBT does not ensure definitive liberation from mechanical ventilation [6, 12, 14]. Systematic reviews and large observational studies have consistently reported reintubation rates ranging from 15% to 25% within the first 48–72 h after extubation [6, 12, 14]. Early reintubation is strongly associated with increased ICU length of stay, higher mortality, and worse functional outcomes, highlighting the clinical relevance of identifying modifiable determinants of weaning failure [6, 14]. Insufficient inspiratory muscle reserves and diaphragmatic dysfunction have been repeatedly identified as the central pathophysiological mechanisms underlying weaning and extubation failure, particularly in patients exposed to prolonged mechanical ventilation [4–6, 9].

Therefore, inspiratory muscle training (IMT) has emerged as a targeted rehabilitative intervention designed to improve diaphragmatic and accessory inspiratory muscle strength, endurance, and neuromuscular efficiency during weaning process [8, 15, 16]. IMT is considered an anaerobic exercise modality that can be delivered using resistive or threshold loading devices, or by adjusting ventilator sensitivity to impose an inspiratory load [8, 15, 16]. Threshold loading devices are of particular interest because they provide constant, flow-independent inspiratory resistance, ensure a reproducible training stimulus, and potentially offer physiological advantages over variable resistive methods in critically ill patients with unstable breathing patterns [5, 8, 16].

From a physiological standpoint, IMT aims to increase maximal inspiratory pressure (MIP), reduce the rapid shallow breathing index (RSBI; respiratory frequency divided by tidal volume, f/VT), and improve the capacity of the inspiratory muscles to tolerate respiratory load during spontaneous breathing [9, 15, 17]. Randomized controlled trials have shown that IMT can increase MIP, improve tidal volume, and enhance respiratory muscle pressure-generating capacity in mechanically ventilated patients [4, 14–21]. Improvements in MIP and RSBI have been consistently associated with a higher probability of successful weaning and extubation in both intubated and tracheostomized ICU populations [5, 9–13].

Despite increasing clinical interest, the effectiveness of IMT in the ICU setting remains uncertain [8, 15]. Previous systematic reviews have reported consistent improvements in inspiratory muscle strength and surrogate physiological outcomes, such as MIP and RSBI, but have yielded inconclusive or inconsistent findings regarding clinically meaningful outcomes, particularly weaning success and duration of mechanical ventilation [8, 15]. These uncertainties are largely attributable to small sample sizes, heterogeneity in IMT protocols (including intensity, timing, and modality), and reliance on pairwise meta-analyses, which limits the ability to simultaneously compare different IMT strategies [4, 8, 15].

Therefore, a comprehensive synthesis using network meta-analytic methods is warranted to simultaneously compare different inspiratory muscle training modalities and intensities against standard care and alternative respiratory physiotherapy approaches [22].

This systematic review and network meta-analysis aimed to determine whether inspiratory muscle training improves the rate of weaning success in adult ICU patients undergoing mechanical ventilation compared with standard care or no respiratory training. In addition, we evaluated whether IMT significantly improved maximal inspiratory pressure (MIP) and reduced the rapid shallow breathing index (RSBI) at weaning.

We hypothesize that:

- Inspiratory muscle training increases the probability of successful weaning.
- Inspiratory muscle training increases MIP and decreases RSBI compared with control conditions.

## Methods

This systematic review was conducted in accordance with the Preferred Reporting Items for Systematic Reviews and Meta-Analyses (PRISMA) 2020 guidelines [22]. The review protocol was registered in the International Prospective Register of Systematic Reviews (PROSPERO) under registration number CRD420251026681 and developed in accordance with the PRISMA-P recommendations [23]. The methodology adhered to prespecified eligibility criteria, comprehensive database searching, dual independent screening, and rigorous quality assessment.

### Eligibility criteria

We included randomized controlled trials (RCTs) involving adult patients (≥ 18 years) admitted to an intensive care unit (ICU) and receiving invasive mechanical ventilation (IMV) for at least 48 h. Studies were considered eligible if they evaluated the effects of inspiratory muscle training (IMT) delivered via resistive loading, threshold pressure training, or ventilator sensitivity adjustment, compared with conventional care (e.g., spontaneous breathing via T-piece, routine physiotherapy, or no specific respiratory training).

A detailed definition of the intervention categories and network node classifications is provided in Supplementary Table 7.

Eligible studies were required to report at least one of the following outcome measures:

- Weaning success, defined as sustained spontaneous breathing for ≥ 48 h following extubation
- Maximal inspiratory pressure (MIP), assessed at baseline and/or post-intervention
- Rapid shallow breathing index (RSBI), measured during the weaning process.

Studies exclusively enrolling patients with acute COVID-19–related respiratory failure during the pandemic ICU phase were excluded to preserve clinical and pathophysiological homogeneity. Acute COVID-19 ICU populations are characterized by distinct mechanisms of respiratory failure, ventilatory strategies, sedation practices, and inflammatory profiles, which may independently affect diaphragmatic function and weaning outcomes. Studies enrolling post-COVID mechanically ventilated patients after resolution of the acute infectious phase were considered eligible, as these populations resemble non-COVID critically ill patients in terms of ventilatory management and respiratory muscle dysfunction. Studies were excluded if they enrolled pediatric patients, did not meet the RCT design criteria, or did not report extractable data for the outcomes of interest.

Invasive mechanical ventilation was defined as ventilatory support delivered via an endotracheal tube or a tracheostomy. Tracheostomized patients were included if they remained dependent on invasive ventilatory support, as they represent a clinically relevant subgroup of prolonged or difficult-to-wean ICU patients. From a pathophysiological perspective, inspiratory muscle dysfunction and ventilator dependence persist, regardless of airway access. Therefore, these populations were considered part of the same weaning continuum in this analysis. Studies were eligible regardless of the timing of IMT initiation provided that training was delivered during invasive mechanical ventilation, during the weaning phase, or immediately after extubation in patients recently liberated from invasive ventilation. This inclusive approach was adopted to reflect real-world clinical practice and capture the full spectrum of IMT applications across the different phases of the weaning continuum.

For network meta-analysis classification purposes, IMT intensity was categorized a priori. High-intensity IMT (HI-IMT) was defined as the use of ≥ 30% of maximal inspiratory pressure (MIP), typically involving progressive overload or target loads of ≥ 40% MIP during training sessions. Low-intensity IMT (Lo-IMT) was defined as the use of ≤ 10% MIP or sham loading strategies that were not intended to induce a meaningful training stimulus.

### Information sources and search strategy

An initial literature search was conducted in PubMed, Cochrane Library, and PEDro through July 28, 2025, by two senior reviewers (E.M.-O. and E.A.S.R.). All search strategies were constructed using combinations of Medical Subject Headings (MeSH) and non-MeSH terms and adapted to the indexing system of each database related to inspiratory muscle training, mechanical ventilation, weaning, and physiological outcomes (MIP and RSBI). Boolean operators (AND/OR) were applied to appropriately combine terms. Only the studies published in English or Spanish were included.

- Detailed search strings for each database, including the refined PubMed strategy, are provided in Supplementary Material 1.
  - ("Inspiratory Muscle Training"[tiab] OR "IMT"[tiab] OR "Respiratory Muscle Training"[tiab])
  - AND ("Weaning"[MeSH] OR "Ventilator Weaning"[tiab] OR "Extubation"[tiab])
  - AND ("Mechanical Ventilation"[MeSH] OR "Critical Care"[MeSH] OR "ICU"[tiab])
  - AND ("Maximal Inspiratory Pressure"[tiab] OR "MIP"[tiab] OR "Rapid Shallow
  - Breathing Index"[tiab] OR "RSBI"[tiab] OR "f/VT"[tiab])AND ("Randomized Controlled Trial"[pt] OR "RCT"[tiab])
- Equivalent strategies, adapted to their indexing and filtering capabilities, were used for the Cochrane Library and PEDro.

The reference lists of included studies and relevant reviews were manually screened to identify additional eligible trials.

### Selection process

Two senior reviewers (E.M.-O. and E.A.S.R.) independently screened the titles and abstracts retrieved from all databases. Disagreements were resolved by discussion or consultation with a third senior reviewer (J.N.C.Z.).

Eligible full texts were independently assessed by both the reviewers. The final inclusion list was based on consensus.

### Data collection and extraction

Data extraction was performed independently by two researchers (C.C. and N.C.C.) using a standardized form. The following data were collected.

- Study characteristics: authorship, year, country, study design, sample size
- Participant characteristics: age, sex, clinical status, MV duration
- Intervention details: type of IMT (device, load, frequency, duration)
- Control group characteristics
- Outcomes: weaning success, MIP, RSBI (including timepoints and results).

Any discrepancies in data extraction were resolved through discussion.

### Risk of bias assessment

The risk of bias was initially assessed using E.M.-O. and E.A.S.R. using the Cochrane Risk of Bias 2.0 (RoB 2.0) tool for randomized controlled trials [24]. A final independent re-evaluation of all included studies was conducted to ensure consistency and accuracy. This tool evaluates the following five domains.

1. Randomisation process
2. Deviations from intended interventions
3. Missing outcome data
4. Measurement of the outcome
5. Selection of the reported result.

Each domain was rated as “low risk, ‘some concerns,” or “high risk. ’ The overall risk of bias was determined based on domain-level ratings.

### Statistical analysis

The R program (version 4.1.3, R Foundation for Statistical Computing) [25], metafor (version 4.4.0) [26], and netmeta (version 3.2.0) [27] packages were used for statistical analysis.

A frequentist network meta-analysis (NMA) was performed. The standardized mean difference (SMD) was used for both the Maximal Inspiratory Pressure (MIP) and Rapid Shallow Breathing Index (RSBI). SMD was used for MIP because the studies reported it in different units (cmH₂O and percentages). Because several trials reported MIP exclusively as a percentage of predicted values and did not provide sufficient information to allow reliable back-conversion to absolute units (cmH₂O), a common-unit mean difference approach was not feasible across all included studies. Regarding RSBI, although it was measured on a common scale, SMD was preferred because of the significant disparity in the coefficients of variation between studies (ranging from r to coefrsbi), which allowed for the normalization of effects based on specific study dispersion, preventing studies with extreme variances from biasing the overall estimate. Weaning success was analyzed using odds ratios (OR).

In MIP, a sensitivity analysis was performed following the recommendations of Efthimiou et al. [28] among naïve studies and excluding those by Bisset et al. [16, 29] to ensure the robustness of the model. Finally, the IMT groups from Abdeen et al. [30] and Ratti et al. [21] were combined, and the standard deviations were calculated from the confidence intervals in the study by Wang et al. [31] using the formulas recommended in the Cochrane Handbook [32] (Supplementary material Table 1).

The transitivity assumption was evaluated by assuming that all analyzed interventions presented the same results, regardless of the study to which they belonged. The effect modifiers were selected a priori based on their clinical relevance and availability across studies, including age, sex distribution, maximal inspiratory pressure threshold, and weaning duration. To do this, the presence of significant differences between the different interventions in age, male/female ratio, maximal inspiratory pressure percentage threshold and weaning period (days) was analyzed using the Kruskal–Wallis H test, and a meta-regression was performed controlling the model with the same as confounding variables, with the aim of analyzing whether they significantly influence the NMA results and their impact on the level of heterogeneity.

Model heterogeneity and consistency were analyzed using Likelihood Ratio Tests (LRT) and Akaike Information Criterion (AIC). Heterogeneity was assessed by comparing the fixed-effects models with the random-effects models. Network consistency was assessed using a global design-by-treatment interaction model by comparing it to an inconsistent (unrestricted) model. In both cases, the significance level was set at *p* < 0.05, and the lower AIC values were used to identify the best-fitting model. Tests for local inconsistencies, such as node splitting, were not applicable because of insufficient closed loops in the network structure.

Between-study variance was calculated using τ^2^ with the Restricted Maximum Likelihood (REML) estimator, Cochrane’s Q test, and I^2^ estimator, defined as follows: 0%–30%, unimportant heterogeneity; 30%–50%, moderate heterogeneity; 50%-75%: large heterogeneity; 75%-100%: significant heterogeneity [32].

The effectiveness of the interventions was analyzed using the league table for direct comparisons, P-score rankings, and Surface Under the Cumulative Ranking Curve (SUCRA), which was calculated using a resampling method (100 simulations) to ensure a robust ranking estimate. Additionally, visual inspection of the rankograms was performed to confirm the results.

Finally, publication bias was analyzed using a funnel plot adjusted for each comparison, and the Egger, Begg-Mazumbar, and Thompson-Sharp tests were applied.

## Results

### Study selection and characteristics

**Fig. 1:**
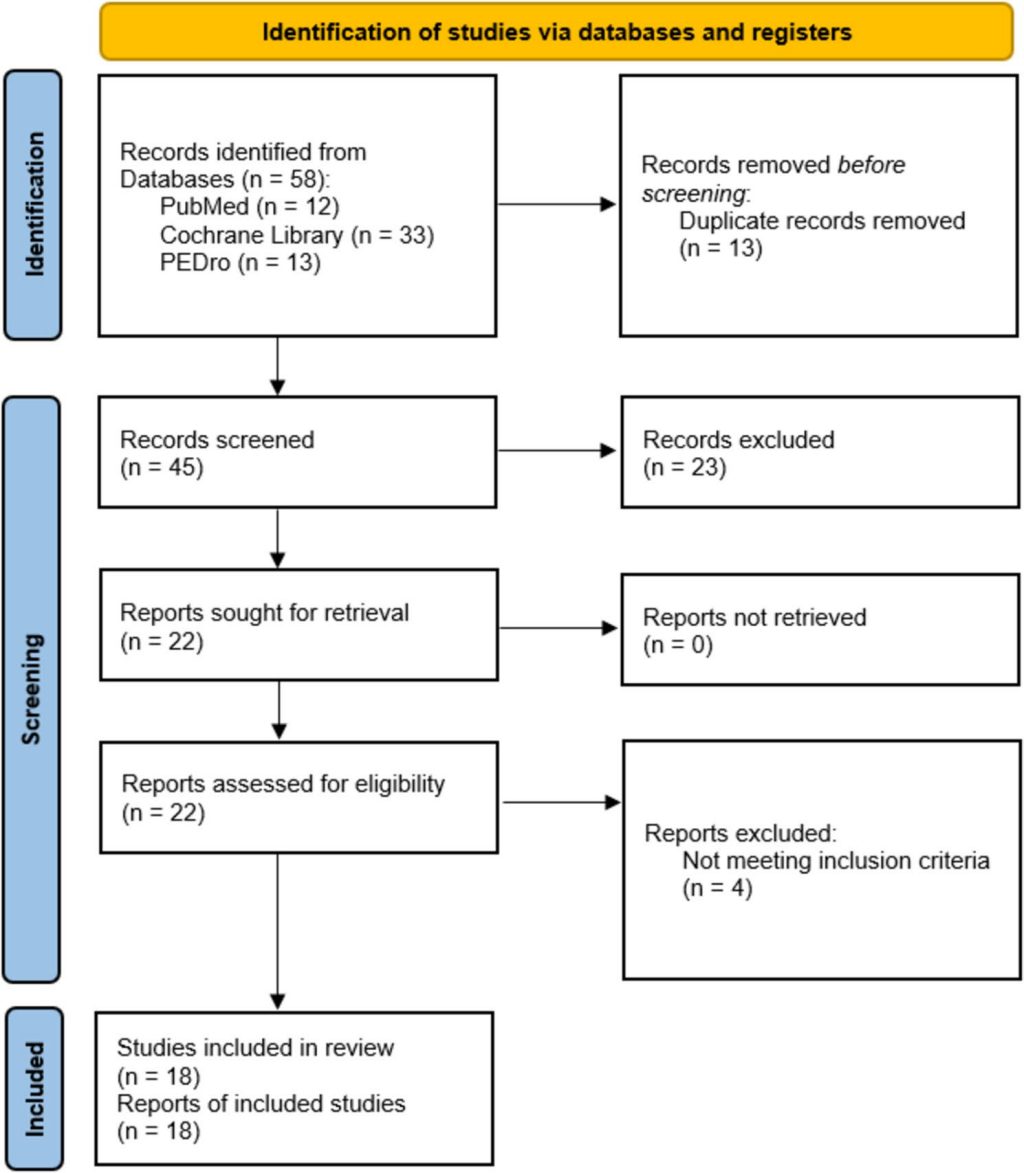
PRISMA flow diagram. Search strategy

A search of electronic databases identified 58 records. After removing 13 duplicates, 45 titles and abstracts were screened. Of these, 27 were discarded due to incompatibility with the inclusion criteria, lack of full-text availability, or incomplete trial status. Ultimately, 18 RCTs met al.l the inclusion criteria and were included in the qualitative synthesis (Fig. 1) [22].

### Characteristics of included studies

A total of 1137 participants from 18 RCTs [14–40] were included in this review. Studies have been conducted in various countries including Brazil, India, Australia, Colombia, Belgium, Turkey, China, Taiwan, Egypt, Pakistan, Iran, and France. All trials included adult ICU patients undergoing invasive mechanical ventilation for at least 48 h (Table 1).

**Table 1:** Characteristics of the randomized controlled trials included in the systematic review

Author (Year)	Country	Study Design	Population	IMT Modality	Comparator	Outcomes Reported	Main Findings
Caruso et al. (2005)	Brazil	RCT	ICU adults (*n* = 25)	HI-IMT (40% MIP, 2×/day)	Usual care	MIP, MV duration	Significant ↑ in MIP; ↓ MV duration
Cader et al. (2010)	Brazil	RCT	Elderly ICU patients (*n* = 28)	HI-IMT (30% MIP, + 10% daily, 2×/day)	Usual care	MIP, RSBI, weaning time	Significant ↑ in MIP & RSBI; ↓ weaning time
Robledo Condessa et al. (2013)	Brazil	RCT	ICU adults (*n* = 77)	HI-IMT (40% MIP, 2×/day)	Usual care	MIP, RSBI, TV, weaning time	Significant ↑ in MIP & TV; no significant change in weaning time
Dixit & Prakash (2014)	India	RCT	ICU adults (*n* = 30)	HI-IMT (30% MIP, 2×/day)	URPth	MIP, weaning time	Significant ↑ in MIP; ↓ weaning time
Bissett et al. (2016)	Australia	RCT	ICU survivors post-extubation (*n* = 70)	HI-IMT (50% MIP, 2 wks)	URPth	MIP, FRI	Significant ↑ in MIP; no significant change in FRI
Sandoval Moreno et al. (2019)	Colombia	RCT	ICU adults with ARF (*n* = 102)	HI-IMT (50% MIP, 2×/day)	URPth	MIP, weaning time/success	MIP ↑ slightly (NS); weaning time and success ≈ no difference
da Silva Guimarães et al. (2021)	Brazil	RCT	ICU patients after cardiac surgery (*n* = 101)	HI-IMT (40% MIP, 2×/day)	URPth	MIP, inspiratory effort index, weaning success, ICU survival	Significant ↑ in MIP, inspiratory effort index & weaning success; ↑ ICU survival
Roceto Ratti et al. (2022)	Brazil	RCT	Tracheostomized ICU pts (*n* = 104)	HI-IMT (30% PImax, 2×/day)	URPth	MIP, RSBI, weaning time, weaning success, ICU stay, VM duration	Significant ↑ in MIP; no significant change in weaning outcomes, RSBI, ICU or VM duration
Van Hollebeke et al. (2022)	Belgium	RCT	Weaning-difficult ICU pts (*n* = 69)	HI-IMT (30–50% PImax, daily, 28d)	Control: Sham IMT (10% PImax)	MIP, FVC, PIF	Significant ↑ in MIP, FVC & PIF (only in high-intensity IMT)
Bissett et al. (2023)	Australia	RCT	ICU patients on MV ≥ 7 days (*n* = 70)	HI-IMT (50% MIP, 5 × 6 reps)	URPth	MIP, FRI, VM duration, QoL, dyspnoea	NS ↑MIP & VM duration; significant ↑FRI & QoL; ↓dyspnoea
Benli et al. (2024)	Turkey	RCT	Post-extubation ICU pts (*n* = 20)	HI-IMT (PowerBreathe, 30% MIP)	URPth	MIP, diaphragm excursion	↑MIP, ↑DE in IMT group
Reginault et al. (2024)	France	RCT	Difficult-to-wean ICU pts (*n* = 89)	HI-IMT (40% MIP, 2×/day)	HI-strength IMT (≥ 9 cmH₂O) Mixed- HI-IMT (30–60% MIP)	MIP, weaning success, weaning time (MV duration)	↑MIP in all groups; no significant differences in weaning success/time across modalities”
Zhou et al. (2024)	China	RCT	Neurocritical ICU pts (*n* = 47)	HI-IMT + PNF (50% MIP, 2×/day, 5 days/week)	IMT only	MIP, DE	Significant ↑MIP & DE in intervention group
Kazemi et al. (2024)	Iran	RCT	ICU adults (*n* = 70)	HI-IMT + URPth (40% MIP, 2x/day, 1wk)	URPth	MIP, weaning/extubation success & time, DE	Significant ↑MIP & extubation success; ↓extubation time; ↑DE
Iqbal et al. (2024)	Pakistan	RCT	Post-COVID MV patients (*n* = 22)	HI-IMT (50% PMmax, 30 breaths, 2×/day, 4 weeks)	Sham IMT (10% PMmax, 1×/day)	MIP, FEV1/FVC, CKM	Significant ↑MIP & FEV1/FVC; ↓CKM in intervention group
Abdeen et al. (2025)	Egypt	RCT	ICU adults (*n* = 90)	HI-IMT (30% MIP, 2×/day)	URPth	MIP, RSBI, weaning success	Significant ↑MIP & ↓RSBI; higher weaning success
Wang et al. (2025)	Taiwan	RCT	Subacute ICU patients (*n* = 33)	HI-IMT (30–50% MIP, 2×/day, 3 wks)	Usual care	MIP, RSBI, MV duration	Significant ↑MIP & ↓RSBI; shorter weaning time
Van Hollebeke et al. (2025)	Belgium	RCT	ICU patients on MV ≥ 48 h (*n* = 90)	High-intensity IMT (≥ 60% MIP)	Lo-IMT (sham, < 10% MIP)	MIP, RSBI, weaning success/time	Significant ↑MIP & ↑weaning success; ↓weaning time; RSBI ≈ no change

Each included study examined the effect of inspiratory muscle training (IMT) on adult ICU patients requiring mechanical ventilation (MV) for at least 48 h. This table summarizes the key methodological characteristics, IMT protocols, comparators, outcome measures, and principal findings. All studies were randomized controlled trials (RCTs) and contributed data for qualitative synthesis and meta-analysis. The main findings are reported descriptively, as stated by the original authors, and do not necessarily reflect the results of the network meta-analysis. Only outcomes related to weaning success, maximal inspiratory pressure (MIP), and rapid shallow breathing index (RSBI) were included in the quantitative synthesis

*Abbreviations*: *ARF* Acute respiratory failure, *DTF* Diaphragmatic thickening fraction, *DE* Diaphragm excursion, *FEV₁/FVC* Forced Expiratory Volume in 1 second/Forced Vital Capacity, *FRI* Fatigue Resistance Index, *HI* High intensity, *ICU* Intensive care unit, *IMT* Inspiratory muscle training, *Lo* Low, *MIP* Maximal inspiratory pressure, *MV* Mechanical ventilation, *NS* Not significant, *RSBI* Rapid shallow breathing index, *PEP* Positive expiratory pressure, *PIF* Peak Inspiratory Flow, *QoL* Quality of life, *PMmax* Maximal pressure of inspiratory muscles, *TV* Tidal volume, *URPth* Usual respiratory physiotherapy. PImax and PMmax were considered to be equivalent to MIP for synthesis. *Symbols*: ↑ increase, ↓ decrease

IMT protocols varied across studies, including:

- Threshold IMT at 30–50% of Maximal Inspiratory Pressure (MIP) [5, 14, 18, 19, 29, 30,33, 35, 37]
- High-intensity protocols at or above 60% MIP [38]
- PowerBreathe devices [21, 39]
- Combination with positive expiratory pressure (PEP) [34, 40] or proprioceptive neuromuscular facilitation (PNF) [40].

Comparators included usual care, sham IMT, conventional chest physiotherapy, or other IMT protocols. Most studies reported outcomes such as MIP (17/18), RSBI (Rapid Shallow Breathing Index) (10/18), weaning success (10/18), duration of mechanical ventilation, or hospital stay. For the detailed characteristics, see Table 1.

### Risk of bias

**Fig. 2:**
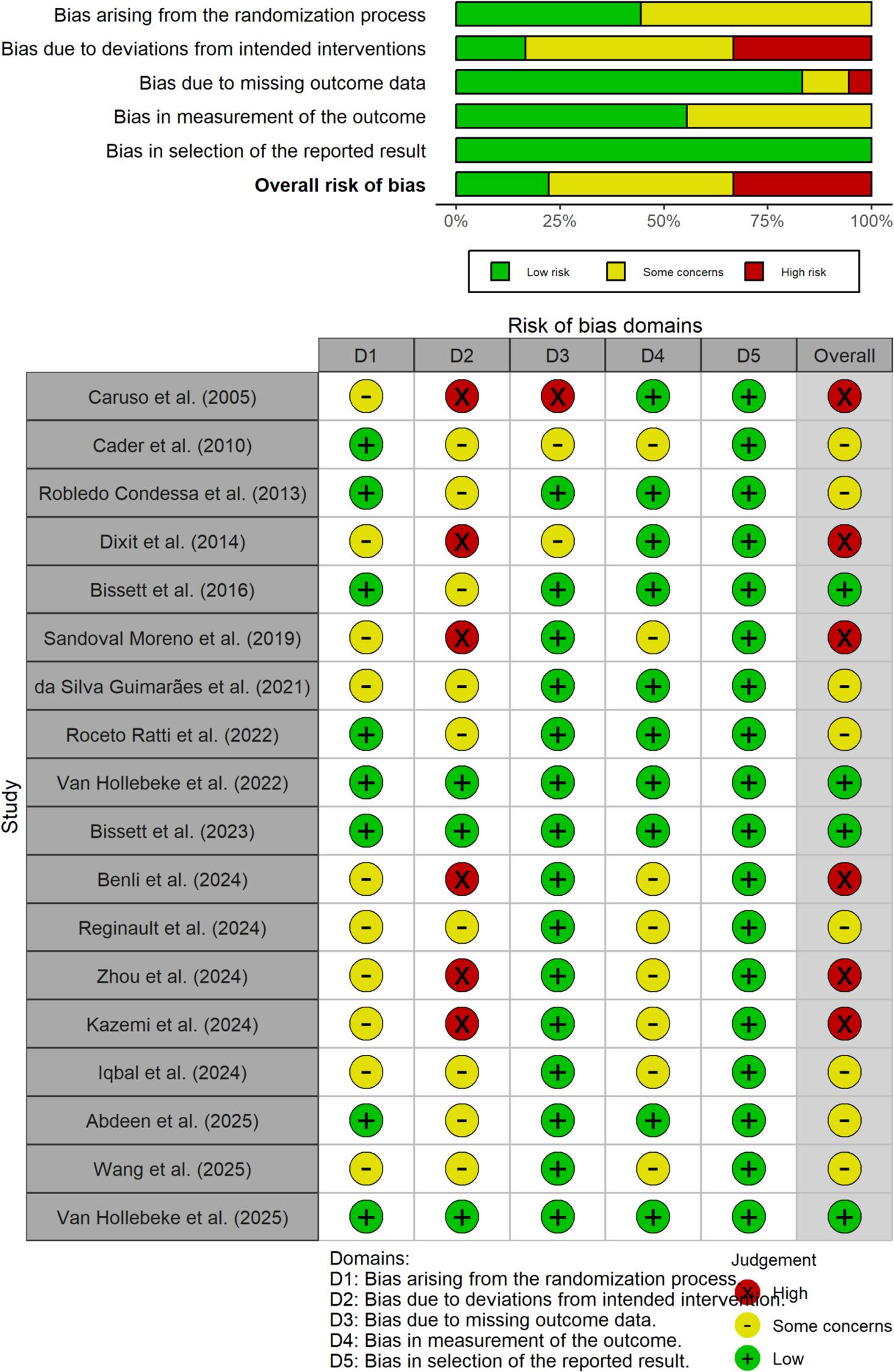
Risk of bias tool for randomized trials (RoB2)

All studies were assessed using the Cochrane RoB 2.0 [24]. As shown in Fig. 2, among the 18 included studies, 4 were rated as having *low risk of bias*, 8 were classified as having *some concerns*, and 6 were judged to have *high risk of bias* overall. The main limitations were identified in the randomization process (domain 1) and deviations from the intended interventions (domain 2). In contrast, the domains related to missing outcome data (Domain 3), outcome measurement (Domain 4), and selection of reported results (Domain 5) showed a low risk of bias across most studies.

### Summary of main outcomes

#### Weaning success

Ten studies [19–21, 30, 36, 38–5] assessed the effect of IMT on weaning success. While seven studies reported improved weaning rates with IMT, only four showed statistically significant differences compared to controls [4, 5, 19, 30]

#### Maximal inspiratory pressure (MIP)

Seventeen trials [14–21, 30–35, 37–40] evaluated the MIP. Most studies have found statistically significant increases in MIP following IMT, including trials with elderly or high-risk populations [5–40]. One study [29] showed a non-significant change in MIP despite improvement in symptoms.

#### Rapid shallow breathing index (RSBI)

Although 10 studies reported RSBI [5, 14–19, 30, 33–36, 38], only six provided sufficient and methodologically comparable data to be included in the network meta-analysis. IMT was associated with a significant reduction in RSBI in 8 patients, indicating improved respiratory efficiency [5, 14, 18, 19, 30, 34–36].

#### Other outcomes

Some studies have reported additional benefits, including improved diaphragm thickness or excursion [34, 39], decreased levels of muscle damage biomarkers [37], better quality of life [16, 29], and improved ₂/FiO₂ ratio [40]

### Quantitative analysis

Maximal Inspiratory Pressure sensitivity analysis showed that the P-score of the interventions did not change between the naïve studies and the studies by Bisset et al. (2016, 2023), with the order of the three being HI-IMT + PNF, HI-IMT, and Lo-IMT (0.934, 0.695, 0.556, and 0.971, 0.687, 0.545, respectively). Furthermore, the meta-analysis did not change the level of significance of the interventions; therefore, all the studies were retained (Supplementary Material Figure 1).

#### Transitivity

The transitivity assumption was assessed by examining the distribution of potential effect modifiers across treatment nodes. The following study-level variables were considered a priori as clinically relevant modifiers: mean age, male-to-female ratio, baseline maximal inspiratory pressure threshold (%MIP), and weaning period duration. Differences across treatment nodes were evaluated using the Kruskal–Wallis test. Although a statistical difference was observed in the maximal inspiratory pressure threshold percentage (*p* = 0.031), subsequent meta-regression analyses demonstrated that none of the covariates significantly influenced the treatment effects. These findings support the plausibility of the transitivity assumption within the network (Supplementary Material Tables 2 and 3).

#### Heterogeneity and consistency

Model selection was based on likelihood ratio tests (LRT) and Akaike Information Criterion (AIC) comparisons between fixed- and random-effects models. For the maximal inspiratory pressure and rapid shallow breathing index, the random-effects model showed a better fit (lower AIC and significant LRT), whereas for weaning success, the fixed-effects model provided a more appropriate fit.

Consistency was evaluated by using a design-by-treatment interaction approach. In this framework, the reduced model corresponds to the consistency model, whereas the full model corresponds to the inconsistency model. Non-significant LRT results and lower AIC values in favor of the reduced model indicated no evidence of inconsistency within the network (Supplementary Material Table 4).

Therefore, the final models selected were those without adjusting for any covariates, applying a random-effects model to the Maximal Inspiratory Pressure, Rapid Shallow Breathing Index, and a fixed-effects model to the variable weaning success.

The choice not to adjust the final network models for covariates was supported by the absence of a statistically significant effect modification in meta-regression analyses and by the adequate balance of potential effect modifiers across treatment nodes.

Substantial heterogeneity was observed for maximal inspiratory pressure (MIP) (Cochrane Q X²(13) = 88.905, *p* < 0.001; I²=85.4%; τ²=0.786). Similarly, rapid shallow breathing index (RSBI) showed considerable heterogeneity (X²(3) = 32.313, *p* < 0.001; I²=90.7%; τ²=0.828). In contrast, heterogeneity was negligible for weaning success (χ ²(10) = 7.381, *p* = 0.689; I²=0%; τ²=0.248). These findings justified the use of random-effects models for MIP and RSBI, and a fixed-effects model for weaning success. The network graph indicated that most indirect comparisons involved short evidence paths (average path length close to two), supporting acceptable network connectivity (Supplementary material Fig. 2).

No direct comparison influenced more than 1% of the total mixed comparisons; therefore, it is unlikely that the methodological quality of the individual articles biased the consistency of the analysis (Supplementary Material Table 5).

#### Network structure

**Fig. 3:**
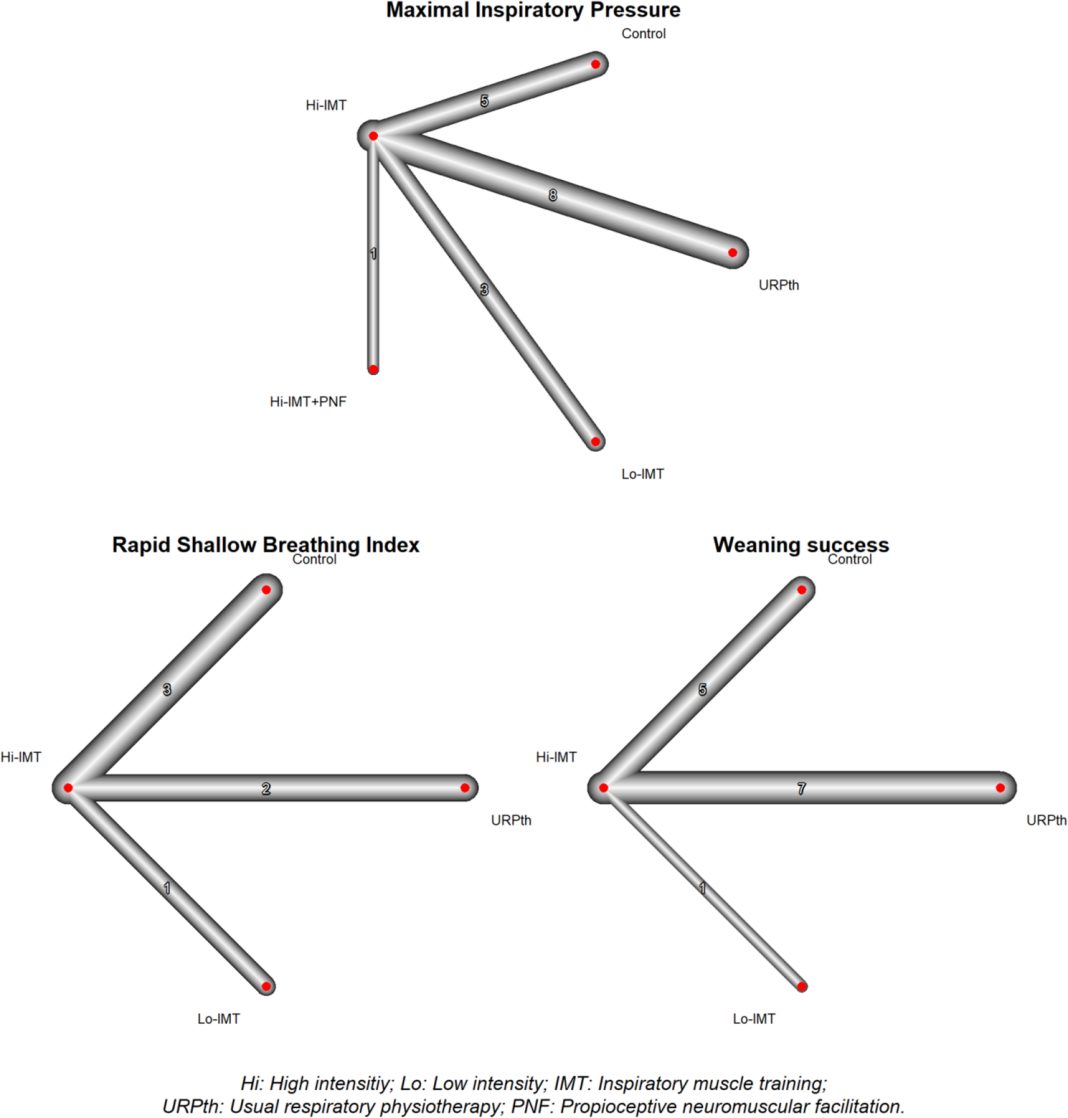
Network comparisons

In the analysis of Maximal Inspiratory capacity, 17 studies featuring four designs with a total of five interventions and 17 pairs of comparisons were included, including a total of 994 patients. For Rapid Shallow Breathing, six studies featuring three designs with a total of four interventions and six pairs of comparisons were included, including a total of 396 patients. Finally, weaning success included 13 studies in which 3 designs with a total of 4 interventions and 13 pairs of comparisons were included, including a total of 814 patients (Supplementary material Table 6). The network graph shows that the largest number of studies compared HI-IMT vs. controls and URPth for all three outcome variables (Fig. 3).

#### Ranking of interventions

The ranking of interventions showed that HI-IMT + PNF, HI-IMT, and Lo-IMT had the greatest impact on Maximal Inspiratory Pressure; HI-IMT and Lo-IMT had the greatest impact on the Rapid Shallow Breathing Index and weaning success (Supplementary Material Figure 3) (Table 2).

**Table 2:** Ranking of interventions based on P-scores and SUCRA values for each outcome in mechanically ventilated ICU patients

		P-score		SUCRA
Maximal Inspiratory Pressure	HI-IMT+PNF	0.934	HI-IMT+PNF	0.948
	HI-IMT	0.695	HI-IMT	0.677
	Lo-IMT	0.556	Lo-IMT	0.565
	Control	0.201	Control	0.208
	URPth	0.114	URPth	0.102
Rapid Shallow Breathing Index	HI-IMT	0.789	HI-IMT	0.800
	Lo-IMT	0.684	Lo-IMT	0.677
	Control	0.287	Control	0.270
	URPth	0.239	URPth	0.253
Weaning success	HI-IMT	0.849	HI-IMT	0.823
	Lo-IMT	0.773	Lo-IMT	0.803
	URPth	0.261	URPth	0.257
	Control	0.117	Control	0.117

Higher P-scores and SUCRA values indicate a higher probability of being the most effective intervention for the corresponding outcome. Rankings were based on the post-intervention outcomes assessed during the weaning phase or immediately after extubation. P-scores were used as the primary ranking metric under the frequentist framework, whereas SUCRA values were calculated using a resampling approach to provide a complementary probabilistic interpretation

*HI* High-intensity, *Lo* Low-intensity, *IMT* Inspiratory muscle training, *URPth* Usual respiratory physiotherapy, *PNF* Proprioceptive neuromuscular facilitation

#### Significant interventions

No differences were found in the Rapid Shallow Breathing Index (SMD) between interventions. In the Maximal Inspiratory Pressure (SMD), higher pressures were observed in the HI-IMT, HI-IMT + PNF (-0.886 (-1.641, -0.131) and − 1.874 (-3.694, -0.054), respectively), HI-IMT compared to the URPth [1.048 (0.465, 1.632] ), and HI-IMT + PNF compared to the URPth [-2.037 (-3.792, -0.281] ) groups than in the control group. In the weaning success outcome (OR), the control group showed a significantly lower probability of successful weaning than the HI-IMT (OR 0.322, 95% CI, 0.138–0.753). Comparisons between control and URPth, as well as between control and Lo-IMT, did not reach statistical significance. HI-IMT demonstrated a significantly higher probability of successful weaning than URPth (OR 2.370, 95% CI 1.478–3.800), whereas other comparisons were not statistically significant (Table 3 and Supplementary Material Figure 4).

**Table 3:**
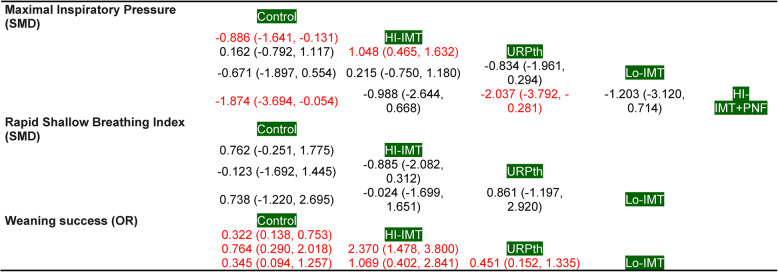
League table reporting the comparative network effects

For continuous outcomes (MIP and RSBI), standardized mean differences (SMDs) lower than zero favored the intervention listed in the row, whereas SMDs greater than zero favored the comparator listed in the column. For weaning success, odds ratios (ORs) greater than 1 favor the intervention listed in the row. Significant differences are shown in red. Comparisons should be read from left to right, with the intervention listed in the row and compared to the intervention in the column

*SMD* Standardized mean difference, *OR* Odds ratio, *HI* High intensity, *Lo* Low intensity, *IMT* Inspiratory muscle training, *URPth* Usual respiratory physiotherapy, *PNF* Proprioceptive neuromuscular facilitation

#### Publication bias

In all three outcome variables, the Egger (t(15) = 1.473, *p* = 0.161, t(4) = 0.142, *p* = 0.894, t(11)=-0.169, *p* = 0.869, Begg-Mazumbar (Z = 1.648, *p* = 0.099, Z=-0.188, *p* = 0.851, Z=-0.244, *p* = 0.807, respectively), and Thompson-Sharp (t(15) = 0.706, *p* = 0.491, t(4) = 0.475, *p* = 0.66, t(11)=-0.138, *p* = 0.892, respectively) tests were not significant. The funnel plots showed that the interventions were distributed asymmetrically around the axis, ultimately indicating the presence of publication bias, as the small number of studies reduced the statistical power of the tests (Supplementary Material Figure 5).

## Discussion

### Summary of main findings

This systematic review and network meta-analysis included 18 randomized controlled trials (RCTs) that evaluated the effects of inspiratory muscle training (IMT) in adult ICU patients undergoing invasive mechanical ventilation for 48 h or more. Overall, IMT was associated with consistent improvements in inspiratory muscle strength and respiratory efficiency, with more variable effects on clinically relevant outcomes such as weaning success.

Network meta-analysis provides additional insights beyond conventional pairwise comparisons, allowing a structured comparison across intervention intensities. High-intensity IMT consistently ranked higher than the control and low-intensity strategies for maximal inspiratory pressure and showed favorable trends for weaning success. In contrast, low-intensity or sham interventions did not demonstrate clinically meaningful benefits, reinforcing the importance of sufficient inspiratory loading to induce physiological adaptation.

Among the included studies, 17 of the 18 trials reported improvements in maximal inspiratory pressure (MIP), with only one trial failing to demonstrate a significant between-group difference [29]. Ten studies assessed the rapid shallow breathing index (RSBI), of which eight reported significant reductions following IMT. In contrast, although 10 studies evaluated weaning success, only four demonstrated statistically significant improvements compared with the control conditions [4, 5, 19, 30]. These findings highlight the well-established physiological benefits of IMT and the heterogeneous and multifactorial nature of the successful liberation from mechanical ventilation.

Beyond individual trials, network meta-analysis provides an integrative comparative perspective. For MIP, significant between-intervention differences were identified, with HI-IMT combined with proprioceptive neuromuscular facilitation (HI-IMT + PNF) and HI-IMT alone showing superior effects compared with control and usual respiratory physiotherapy (URPth). Ranking analyses consistently identified HI-IMT + PNF and HI-IMT as the most effective interventions for improving inspiratory muscle strength, suggesting clear dose- and modality-dependent effects.

Although high-intensity IMT ranked the highest across several outcomes, it is important to recognize that some of these estimates relied on indirect comparisons and a limited number of direct head-to-head trials. Consequently, the magnitude of the observed differences should be interpreted with caution.

Population characteristics may also partly explain the variability across studies. Several trials have included tracheostomized or prolonged mechanically ventilated patients, who represent a more severe and difficult-to-wean subgroup. These patients may derive greater physiological benefits from inspiratory muscle strengthening, although their clinical outcomes may be influenced by multiple systemic factors beyond respiratory muscle performance.

The timing of IMT initiation may further influence clinical responsiveness. Although most trials initiated IMT during invasive mechanical ventilation or active weaning, some included early post-extubation populations. Although physiological benefits appear consistent across phases, their impact on weaning success may differ depending on whether training is delivered before or after liberation from ventilatory support.

For RSBI, no statistically significant differences were detected between active interventions in the network estimates, although ranking analyses favored HI-IMT and Lo-IMT, indicating potential clinical relevance despite overlapping confidence intervals. Regarding weaning success, HI-IMT ranked the highest, with the league table demonstrating a significantly lower probability of successful weaning in the control group than in the HI-IMT (OR 0.32, 95% CI 0.14–0.75). Although this comparison reached statistical significance, the confidence intervals remained wide, and precision was limited by the available number of direct comparisons.

Importantly, heterogeneity was substantial for MIP and RSBI outcomes (I² > 85%), reflecting variability in IMT protocols, patient characteristics, and timing of outcome assessment. This degree of heterogeneity likely reflects differences in IMT dosing strategies, baseline patient severity, and timing of intervention initiation rather than inconsistencies in the direction of the effect. In contrast, the heterogeneity in weaning success was negligible, suggesting more homogeneous effects across studies for this binary outcome.

Physiologically, these findings are consistent with the current understanding that IMT enhances inspiratory muscle pressure-generating capacity; however, successful weaning depends on a complex interaction between respiratory load, neural drive, diaphragm integrity, sedation practices, and overall systemic recovery [6, 7]. Excessive unloading or respiratory effort may contribute to diaphragmatic injury and weaning failure, limiting the translation of isolated muscle strength gains to consistent clinical success.

Recent physiological studies have demonstrated a load-dependent increase in oxygen consumption during IMT, confirming that high-intensity protocols provide a true training stimulus to inspiratory muscles [10, 41]. Baseline inspiratory strength and applied training load likely modulated responsiveness, which may partly explain the observed differences in effectiveness across IMT modalities and intensities.

### Comparison with previous reviews

Our findings are broadly consistent with those of previous systematic reviews and extend the available evidence. Elkins and Dentice [8] first reported that IMT may facilitate weaning, although their review included only six RCTs and did not allow for comparative effectiveness analysis. Subsequent narrative and systematic reviews emphasized improvements in inspiratory muscle strength but reported inconsistent or uncertain effects on weaning outcomes [42–45].

More recently, Bissett et al. [42] identified moderate-quality evidence supporting the use of IMT to improve inspiratory strength with less certainty regarding clinical outcomes. The present review expands this evidence base by incorporating 18 RCTs and applying a frequentist network meta-analysis, allowing indirect comparisons between IMT intensities, devices, and combined approaches. Importantly, our results suggest that higher-intensity and multimodal IMT strategies are more likely to yield clinically relevant physiological benefits, although these do not uniformly translate into improved weaning success.

### Strengths and limitations

This review adhered to the PRISMA 2020 recommendations and applied the Cochrane RoB 2.0. The use of a frequentist network meta-analysis allowed for the simultaneous comparison of multiple IMT strategies and represents a key methodological strength.

However, this study has several limitations. First, substantial heterogeneity in IMT protocols (intensity, device, duration, and timing) likely influenced the pooled estimates. Second, the definitions and timing of weaning success varied across studies. Third, several trials were small, limiting the statistical power. Fourth, although formal tests for publication bias were not significant, funnel plot asymmetry suggested that publication bias cannot be fully excluded, particularly given the small number of studies that contributed to some comparisons. Finally, the absence of a GRADE assessment limits the formal certainty grading of the evidence.

Additionally, the relatively small number of studies contributing to network comparisons may limit the precision of the indirect estimates.

The inclusion of tracheostomized patients reflects real-world IMT application but may have contributed to clinical heterogeneity across studies.

### Implications for practice and research

Current evidence supports IMT as an effective adjunctive therapy to improve inspiratory muscle strength and respiratory efficiency in mechanically ventilated ICU patients. High-intensity IMT, particularly when combined with adjunctive techniques, such as PNF, appears to offer the greatest physiological benefit and may be most appropriate for patients at risk of ventilator-induced diaphragmatic dysfunction or prolonged weaning.

However, the translation of physiological improvements into consistent gains in weaning success remains unclear. Future trials should prioritize standardized IMT protocols, clearly defined weaning outcomes, and patient-centered endpoints such as extubation failure, ICU length of stay, and long-term functional recovery. Therefore, direct head-to-head comparisons of IMT intensity and modality are required.

### Quantitative synthesis

Network meta-analysis clarifies that IMT, especially at higher intensities, produces meaningful improvements in inspiratory muscle strength, with modest and inconsistent effects on weaning success. Ranking analyses consistently favored HI-IMT and HI-IMT + PNF, supporting their prioritization in future clinical trials. However, the substantial heterogeneity and reliance on indirect evidence for some comparisons underscores the need for cautious interpretation.

Overall, these findings reinforce that IMT enhances respiratory muscle function but should be viewed as a component of a multimodal weaning strategy rather than a standalone solution.

## Conclusions

Inspiratory muscle training improves inspiratory muscle strength and respiratory efficiency in mechanically ventilated ICU patients, as demonstrated by significant gains in maximal inspiratory pressure and favorable rankings across interventions. High-intensity IMT has the greatest potential physiological benefits, particularly when combined with adjunctive neuromuscular techniques.

However, the effect of IMT on successful ventilator weaning remains variable, and is influenced by multiple clinical factors. Therefore, IMT should be considered a promising adjunct to conventional weaning strategies rather than a definitive intervention. Well-designed, adequately powered, multicenter RCTs with standardized protocols and patient-centered outcomes are required to clarify their true clinical value.

## Supplementary Information

Supplementary Material 1.

Supplementary Material 2.

## Data Availability

The data presented in this study are available on request from the corresponding authors.

## Abbreviations

ARF: Acute respiratory failure
CPAP: Continuous positive airway pressure
DE: Diaphragm excursion
DTF: Diaphragmatic thickening fraction
FEV_1_: Forced expiratory volume in one second
FVC: Forced vital capacity
FRI: Fatigue resistance index
HI: High intensity
High intensity: High-intensity inspiratory muscle training
ICU: Intensive care unit
IMT: Inspiratory muscle training
IMV: Invasive mechanical ventilation
Lo: Low intensity
Lo-IMT: Low-intensity inspiratory muscle training
MIP: Maximal inspiratory pressure
MV: Mechanical ventilation
NMA: Network meta-analysis
NS: Not significant
OR: Odds ratio
PEEP: Positive end-expiratory pressure
PEP: Positive expiratory pressure
PIF: Peak inspiratory flow
PMmax: Maximal inspiratory muscle pressure
PNF: Proprioceptive neuromuscular facilitation
PRISMA: Preferred Reporting Items for Systematic Reviews and Meta-Analyses
PSV: Pressure support ventilation
QoL: Quality of life
RCT: Randomized controlled trial
REML: Restricted maximum likelihood
RR: Respiratory rate
RSBI: Rapid shallow breathing index
SBT: Spontaneous breathing trial
SMD: Standardized mean difference
SUCRA: Surface under the cumulative ranking curve
URPth: Usual respiratory physiotherapy
VT: Tidal volume
