# Supplementary material for: Efficacy of inspiratory muscle training on weaning success in mechanically ventilated ICU patients: a systematic review and network meta-analysis of randomized controlled trials
Source: BMC Pulm Med. 2026 Mar 27;26:210. doi: 10.1186/s12890-026-04220-3 (PMC13147862; doi:10.1186/s12890-026-04220-3)

**Supplementary material**

**Supplementary material. Figure 1:** Maximal inspiratory pressure sensitivity analysis.


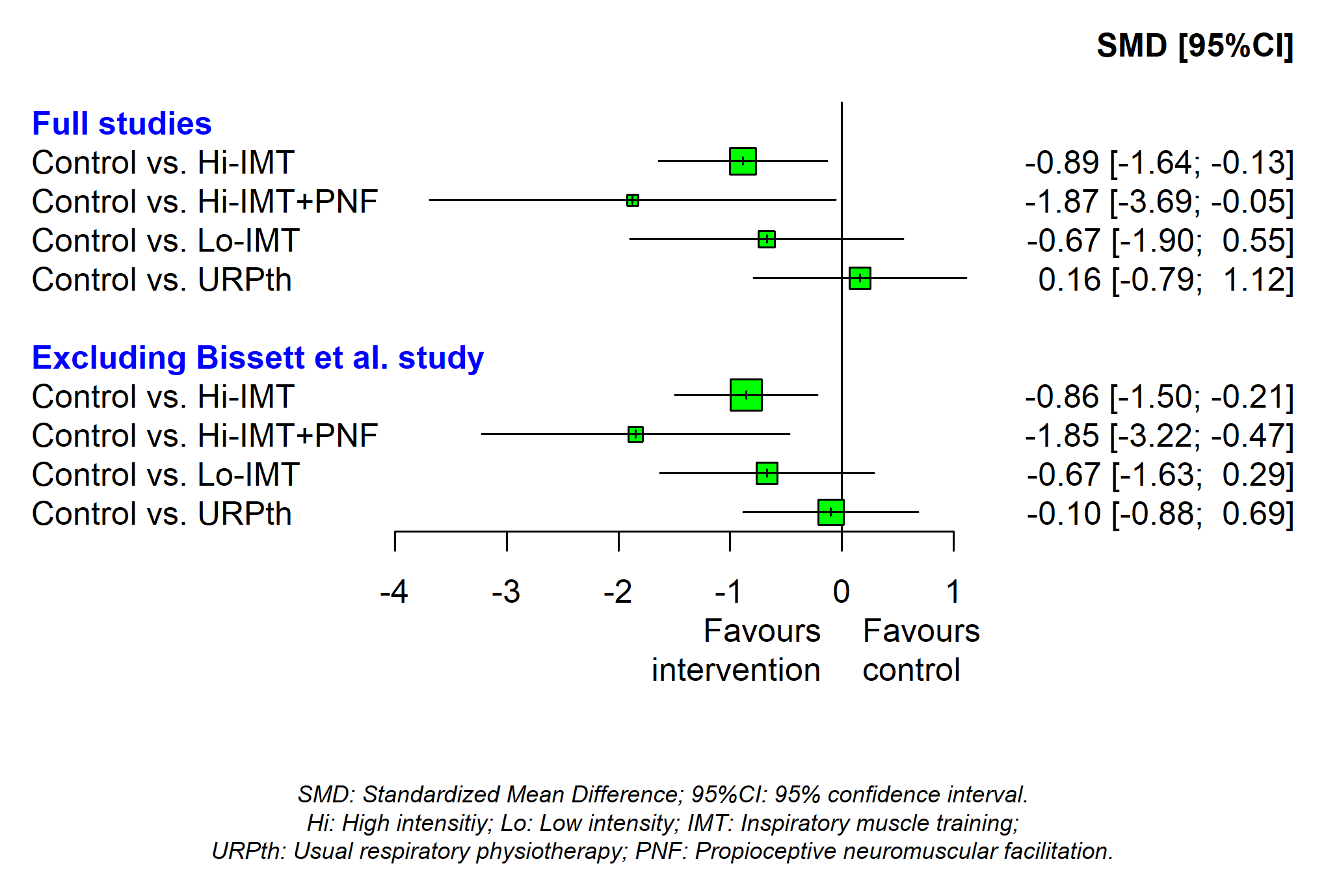


**Supplementary material. Table 1:** Methodological approach for unit standardisation and effect size derivation.

| **Study** | **Reported Data** | **Action Taken** | **Formula/Reference** |
| --- | --- | --- | --- |
| Wang et al. (2025) | 95% CI | Calculated SD | $SD=\frac{Upper-Lower}{3.92}\cdot\sqrt{n}$ |
| Abdeen et al. (2025) / Ratti et al. (2022) | Multiple IMT arms | Combined into one | $SD_{p}=\sqrt{\frac{(n_{1}-1)SD_{1}^{2}+(n_{2}-1)SD_{2}^{2}}{n_{1}+n_{2}-2}}$ |
| Bissett et al. (2016, 2023) | Percentages (%) | Kept as % (SMD analysis) | Sensitivity analysis (Efthimiou) |

n_1_ and n_2_, sample size fo two groups; SD_1_ and SD_2_ standarad deviation of two groups; Upper and Lower 95% confidence interval limits.

**Supplementary material. Table 2:** Comparison of potential effect modifiers across treatment nodes.

| **Variable** | **Control** | **Hi-IMT** | **Hi-IMT+PNF** | **Lo-IMT** | **URPth** | **^a^p value** |
| --- | --- | --- | --- | --- | --- | --- |
| Age | 69.200 | 59.966 | 63.9 | 52.000 | 61.081 | 0.319 |
| Weaning period (days) | 10.127 | 10.936 |  | 37.000 | 8.005 | 0.455 |
| Maximal inspiratory pressure threshold percentage | 38.000 | 37.368 | 50 | 10.000 | 37.500 | 0.031 |
| Male/Female ratio | 1.530 | 1.703 | 1.3 | 1.247 | 2.184 | 0.798 |

Hi: High intensitiy; Lo: Low intensity; IMT: Inspiratory muscle training; URPth: Usual respiratory physiotherapy; PNF: Propioceptive neuromuscular facilitation.

^a^significant if p<0.05 (shown in red).

**Supplementary material. Table 3:**Confounding variables effect.

|  |  | **Coefficient (SE)** | **95%CI** | **Z (^a^p value)** |
| --- | --- | --- | --- | --- |
| **Maximal Inspiratory Pressure** | Age | 0.005 (SE=0.032) | -0.058, 0.068 | Z=0.146, p=0.884 |
|  | Maximal inspiratory pressure threshold percentage | 0.014 (SE=0.026) | -0.037, 0.065 | Z=0.532, p=0.595 |
|  | Male/female ratio | -0.054 (SE=0.347) | -0.734, 0.625 | Z=-0.157, p=0.875 |
|  | Weaning period (days) | -0.007 (SE=0.038) | -0.082, 0.068 | Z=-0.186, p=0.852 |
| **Rapid Shallow Breathing Index** | Age | -0.654 (SE=1.635) | -3.858, 2.55 | Z=-0.4, p=0.689 |
|  | Maximal inspiratory pressure threshold percentage | 1.316 (SE=2.77) | -4.113, 6.744 | Z=0.475, p=0.635 |
|  | Male/female ratio | 30.031 (SE=77.422) | -121.713, 181.776 | Z=0.388, p=0.698 |
|  | Weaning period (days) | -1.195 (SE=2.52) | -6.133, 3.744 | Z=-0.474, p=0.635 |
| **Weaning success*** | Age | 0.993 (SE=0.041) | 0.916, 1.078 | Z=-0.163, p=0.871 |
|  | Maximal inspiratory pressure threshold percentage | 0.998 (SE=0.03) | 0.942, 1.057 | Z=-0.072, p=0.943 |
|  | Male/female ratio | 0.88 (SE=0.318) | 0.434, 1.785 | Z=-0.355, p=0.723 |
|  | Weaning period (days) | 1.015 (SE=0.04) | 0.939, 1.096 | Z=0.371, p=0.711 |

*: Data expressed in odds ratio; SE: Standard error; 95%CI: 95% confidence interval. ^a^significant if p<0.05 (shown in red).

**Supplementary material. Table 4:** Assessment of heterogeneity and consistency.

|  | **Model** | **AIC** | **^a^p value** |
| --- | --- | --- | --- |
| **Heterogeneity test** | | | |
| **Maximal Inspiratory Pressure** | Full | 43 |  |
|  | Reduced | 88 | X^2^(4)=47, p<0.001 |
| **Rapid Shallow Breathing Index** | Full | 34 |  |
|  | Reduced | 36 | X^2^(3)=4, p<0.001 |
| **Weaning success** | Full | 29 |  |
|  | Reduced | 27 | X2(3)<0.001, p>0.999 |
| **Consistency test** | | | |
| **Maximal Inspiratory Pressure** | Full | 45 |  |
|  | Reduced | 43 | X^2^(5)<0.001, p>0.999 |
| **Rapid Shallow Breathing Index** | Full | 36 |  |
|  | Reduced | 34 | X^2^(4)<0.001, p>0.999 |
| **Weaning success** | Full | 31 |  |
|  | Reduced | 29 | X^2^(4)<0.001, p>0.999 |

AIC: Akaike information criterium. ^a^significant if p<0.05 (shown in red).

**Supplementary material. Figure 2:** Direct evidence proportion for each network estimate.


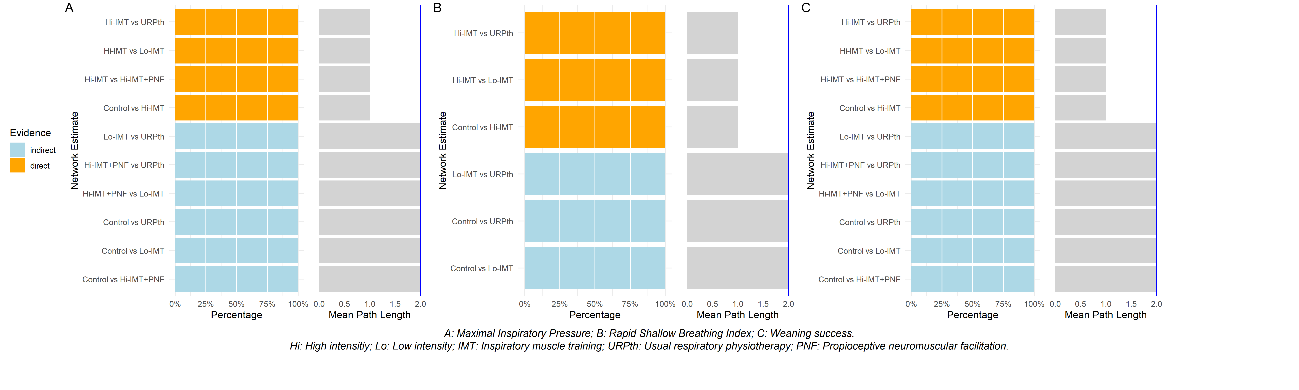


**Supplementary material. Table 5:** Pairwise direct contribution table.

|  |  | **Control vs. Hi-IMT** | **Hi-IMT vs. Hi-IMT+PNF** | **Hi-IMT vs. Lo-IMT** | **Hi-IMT vs. URPth** |
| --- | --- | --- | --- | --- | --- |
| **Maximal Inspiratory Pressure** | Control vs Hi-IMT | >0.999 | <0.001 | <0.001 | <0.001 |
|  | Control vs Hi-IMT+PNF | 0.5 | 0.5 | <0.001 | <0.001 |
|  | Control vs Lo-IMT | 0.5 | <0.001 | 0.5 | <0.001 |
|  | Control vs URPth | 0.5 | <0.001 | <0.001 | 0.5 |
|  | Hi-IMT vs Hi-IMT+PNF | <0.001 | >0.999 | <0.001 | <0.001 |
|  | Hi-IMT vs Lo-IMT | <0.001 | <0.001 | >0.999 | <0.001 |
|  | Hi-IMT vs URPth | <0.001 | <0.001 | <0.001 | >0.999 |
|  | Hi-IMT+PNF vs Lo-IMT | <0.001 | 0.5 | 0.5 | <0.001 |
|  | Hi-IMT+PNF vs URPth | <0.001 | 0.5 | <0.001 | 0.5 |
|  | Lo-IMT vs URPth | <0.001 | <0.001 | 0.5 | 0.5 |
| **Rapid Shallow Breathing Index** | Control vs Hi-IMT | >0.999 |  | <0.001 | <0.001 |
|  | Control vs Lo-IMT | 0.5 |  | 0.5 | <0.001 |
|  | Control vs URPth | 0.5 |  | <0.001 | 0.5 |
|  | Hi-IMT vs Lo-IMT | <0.001 |  | >0.999 | <0.001 |
|  | Hi-IMT vs URPth | <0.001 |  | <0.001 | >0.999 |
|  | Lo-IMT vs URPth | <0.001 |  | 0.5 | 0.5 |
| **Weaning success** | Control vs Hi-IMT | >0.999 |  | <0.001 | <0.001 |
|  | Control vs Lo-IMT | 0.5 |  | 0.5 | <0.001 |
|  | Control vs URPth | 0.5 |  | <0.001 | 0.5 |
|  | Hi-IMT vs Lo-IMT | <0.001 |  | >0.999 | <0.001 |
|  | Hi-IMT vs URPth | <0.001 |  | <0.001 | >0.999 |
|  | Lo-IMT vs URPth | <0.001 |  | 0.5 | 0.5 |

Hi: High intensitiy; Lo: Low intensity; IMT: Inspiratory muscle training; URPth: Usual respiratory physiotherapy; PNF: Propioceptive neuromuscular facilitation. Values higher than 0.8 shown in red.

**Supplementary material. Table 6:** Characteristics of studies.

|  | **Study** | **Comparison** | **Number of participants** | **Effect size±standard error** |
| --- | --- | --- | --- | --- |
| **Maximal Inspiratory Pressure** | Caruso et al. (2005) | Hi-IMT vs Control | 25 | 0.067±0.4 |
|  | Cader et al. (2010) | Hi-IMT vs Control | 28 | 2.412±0.497 |
|  | Robledo Condessa et al. (2013) | Hi-IMT vs Control | 77 | 0.544±0.232 |
|  | Dixit & Prakas. (2014) | Hi-IMT vs URPth | 30 | 1.262±0.4 |
|  | Bissett et al. (2016) | Hi-IMT vs URPth | 70 | 312.416±26.405 |
|  | Sandoval Moreno et al. (2019) | Hi-IMT vs URPth | 102 | 0.237±0.199 |
|  | da Silva Guimarães et al. (2021) | Hi-IMT vs URPth | 101 | 0.696±0.205 |
|  | Roceto Ratti et al. (2022) | URPth vs Hi-IMT | 78 | -0.258±0.229 |
|  | Van Hollebeke et al. (2022) | Hi-IMT vs Lo-IMT | 41 | -0.063±0.313 |
|  | Bissett et al. (2023) | Hi-IMT vs Control | 70 | 0.865±0.25 |
|  | Benli et al. (2024) | Hi-IMT vs URPth | 20 | 0.719±0.461 |
|  | Zhou et al. (2024) | Hi-IMT vs Hi-IMT+PNF | 47 | -0.988±0.309 |
|  | Kazemi et al. (2024) | Hi-IMT vs URPth | 70 | 0.633±0.245 |
|  | Iqbal et al. (2024) | Hi-IMT vs Lo-IMT | 22 | 0.92±0.448 |
|  | Abdeen et al. (2025) | URPth vs Hi-IMT | 90 | -1.66±0.256 |
|  | Wang et al. (2025) | Hi-IMT vs Control | 33 | 0.972±0.368 |
|  | Van Hollebeke et al. (2025) | Hi-IMT vs Lo-IMT | 90 | -0.1±0.211 |
| **Rapid Shallow Breathing Index** | Cader et al. (2010) | Hi-IMT vs Control | 28 | -16.2±4.466 |
|  | Robledo Condessa et al. (2013) | Hi-IMT vs Control | 77 | -12±9.594 |
|  | Roceto Ratti et al. (2022) | URPth vs Hi-IMT | 78 | -0.235±10.363 |
|  | Abdeen et al. (2025) | URPth vs Hi-IMT | 90 | 34.11±4.699 |
|  | Wang et al. (2025) | Hi-IMT vs Control | 33 | -44±17.07 |
|  | Van Hollebeke et al. (2025) | Hi-IMT vs Lo-IMT | 90 | -1±8.754 |
| **Weaning success** | Caruso et al. (2005) | Hi-IMT vs Control | 25 | 0.629±0.877 |
|  | Cader et al. (2010) | Hi-IMT vs Control | 28 | 1±3.034 |
|  | Robledo Condessa et al. (2013) | Hi-IMT vs Control | 77 | 1±3.032 |
|  | Dixit & Prakas. (2014) | Hi-IMT vs URPth | 30 | 0.975±2.987 |
|  | Sandoval Moreno et al. (2019) | Hi-IMT vs URPth | 102 | 1.771±1.099 |
|  | da Silva Guimarães et al. (2021) | Hi-IMT vs URPth | 101 | 1.288±0.433 |
|  | Roceto Ratti et al. (2022) | URPth vs Hi-IMT | 78 | 6.206±1.495 |
|  | Bissett et al. (2023) | Hi-IMT vs Control | 70 | 1.317±0.519 |
|  | Benli et al. (2024) | Hi-IMT vs URPth | 20 | 0±1.118 |
|  | Kazemi et al. (2024) | Hi-IMT vs URPth | 70 | 1.118±0.514 |
|  | Abdeen et al. (2025) | URPth vs Hi-IMT | 90 | 5.324±1.468 |
|  | Wang et al. (2025) | Hi-IMT vs Control | 33 | 1.061±3.09 |
|  | Van Hollebeke et al. (2025) | Hi-IMT vs Lo-IMT | 90 | 0.066±0.499 |

Hi: High intensitiy; Lo: Low intensity; IMT: Inspiratory muscle training; URPth: Usual respiratory physiotherapy; PNF: Propioceptive neuromuscular facilitation.

**Supplementary material. Figure 3:** Rankogram of interventions (SUCRA values).


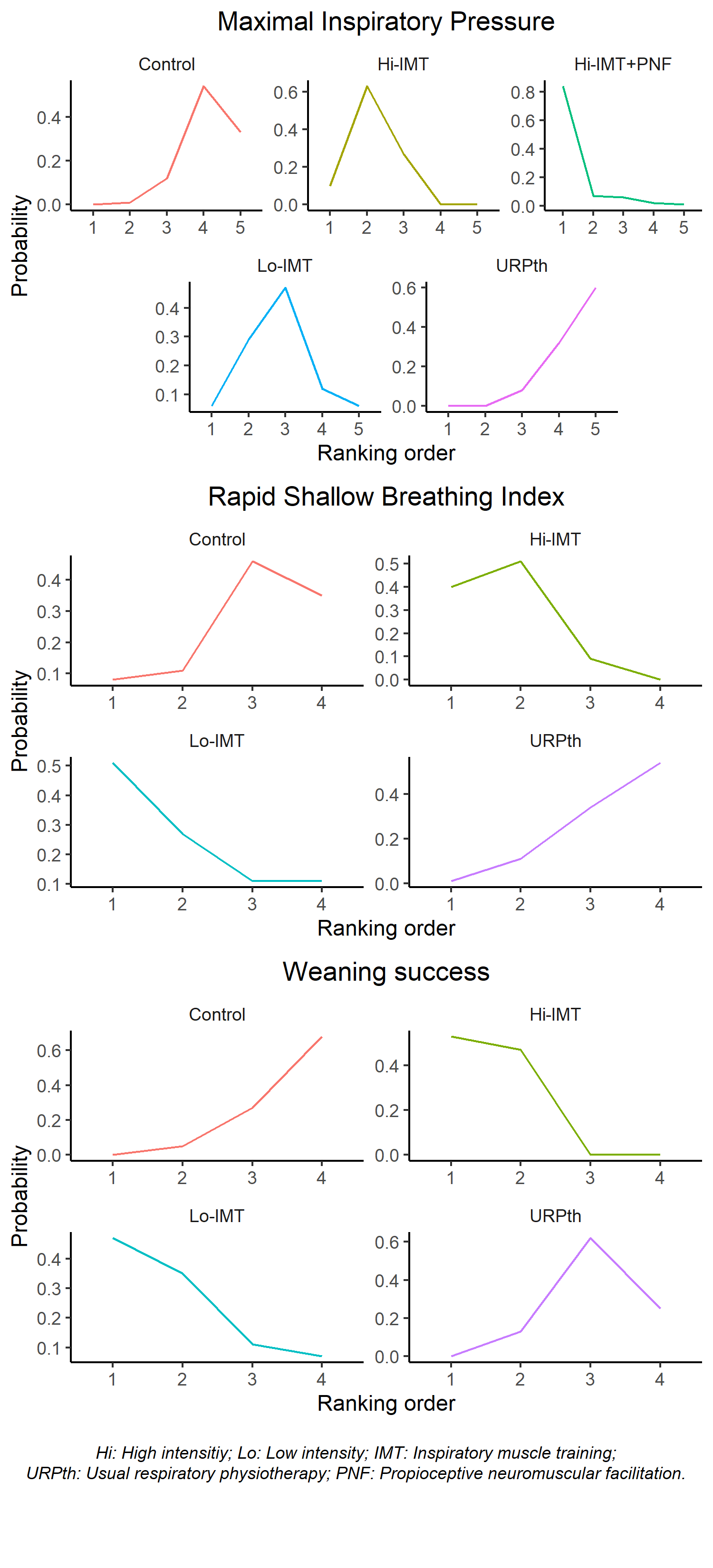


**Supplementary material. Figure 4:** Publication bias funnel plot.


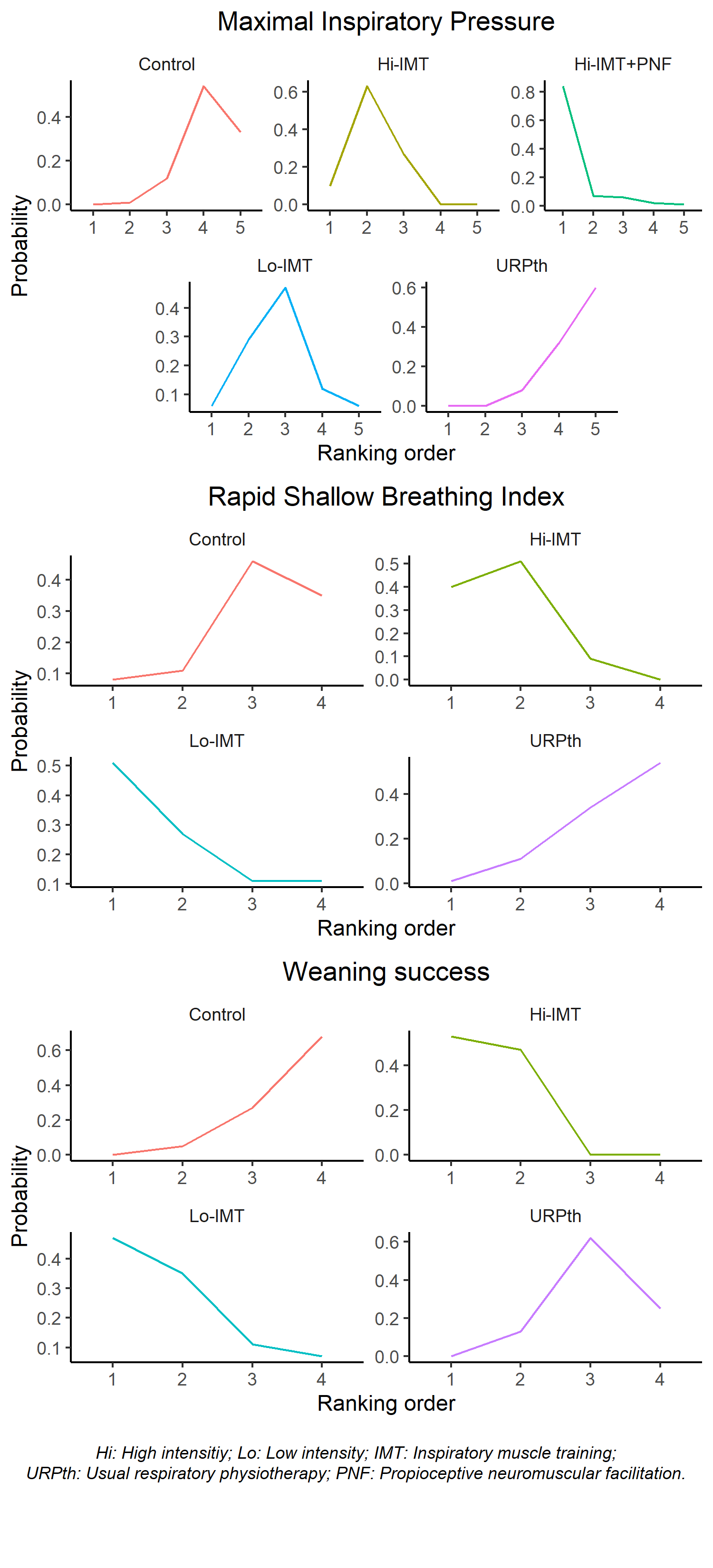

Supplement: Supplementary file 1 — Supplementary Material 1. [file 12890_2026_4220_MOESM1_ESM.docx]
